# CUPRAC Voltammetric Determination of Antioxidant Capacity in Tea Samples by Using Screen-Printed Microelectrodes

**DOI:** 10.1155/2019/8012758

**Published:** 2019-05-14

**Authors:** Alina Elena Trofin, Lucia Carmen Trincă, Elena Ungureanu, Adina Mirela Ariton

**Affiliations:** ^1^Department of Exact Sciences, University of Agricultural Sciences and Veterinary Medicine “Ion Ionescu de la Brad”, Iasi 700490, Romania; ^2^Research and Development Center for Cattle Breeding Dancu, Iasi 707252, Romania

## Abstract

Measurement of antioxidant capacity represents an analytical major challenge in terms of accuracy, efficiency, rapid response, or low cost of detection methods. Quantification of antioxidant capacity of food samples using disposable screen‐printed microelectrodes (SPMEs) was based on cyclic voltammetry versus open-circuit potential (CV vs OCP) and differential pulse voltammetry (DPV) as compared with spectrophotometric measurement of the CUPRAC reaction with 6-hydroxy-2,5,7,8-tetramethylchroman-2-carboxylic acid (trolox). The SPMEs are organic‐resistant electrodes and thus compatible with food samples and organic solvents used to dissolve trolox. A micropipette was used to release a drop of 50 *μ*L sample on the spotted surface of the SPME sensor/working electrode that was time programmed to function according to the working protocol. The SPME response was linearly correlated with trolox content. This preliminary demonstration was focused on the analysis of tea infusions, due to the simplicity and reproducibility of the samples' preparations involved. Analytical results of the antioxidant capacity (expressed as mol·L^−1^ trolox equivalents) of the tea samples showed a good agreement in the case of spectrophotometry and differential pulse voltammetry (*R*^2^ > 0.998). DPV with SPME based on CUPRAC reactions was proven to be a promising approach for the characterization of antioxidant capacity of tea samples with rapid response, cost-effectiveness, and simplicity of operation.

## 1. Introduction

Antioxidants are compounds capable of counteracting the effects of oxidative processes in cells or exogenous systems, reacting in particular with reactive oxygen or nitrogen species [1] or with other free radicals or unstable molecules generated during normal metabolic oxidative reactions [2]. Antioxidant capacity is a general capability of organisms or compounds to engage with free radicals and prevent their harmful effect [3]. Antioxidative effect includes protection of cells and cellular structures against the effect of free radicals, especially oxygen and nitrogen. Antioxidative systems include enzymes and nonenzymatic substrates, such as glutathione, coenzyme Q, vitamins C, A, and E, flavonoids, carotenoids, teine from tea [4–6], and others. Antioxidants are usually found in fruits, vegetables, rice, wine [7], meat, eggs, and other vegetal or animal products, or in food supplements [8].

The search for a simple and accurate rapid method of measuring the antioxidant capacity of a biological sample is a matter of major concern both for fundamental and applicative research as well as for the biotechnological industry. Standard methods employed to evaluate antioxidants from various biological samples are based on colorimetric methods. Different methods have been used to estimate antioxidant capacity, involving 2,2-azino-bis-3-ethylbenzthiazoline-6-sulphonicacid (ABTS) scavenging assays, 1,1-diphenyl-2-picrylhydrazyl (DPPH) radical assays, and ferric-reducing antioxidant power (FRAP) assays [9]. The most common techniques are the colorimetric method of Folin–Ciocalteu for the determination of total phenol content [10], the method determining the oxygen radical absorbance capacity, based on reactions with hydrogen transfer and the method measuring the trolox equivalent antioxidant capacity (TEAC), based on reactions transferring electrons [11–16]. A very reliable method of determining the total antioxidant capacity is the one using the redox system of copper (II)-neocuproine complex (copper-reducing antioxidant capacity method or CUPRAC) [17–23]. Although these methods have been well approved, they are rather complex and time-consuming and use high-priced chemicals or test kits. Also, another limitation of the spectrophotometric methods, especially when analyzing biological samples, is the interference of the biochemical compounds absorbing at the same wavelength as the main reagent, leading to difficulties in precision and accuracy of the results [24].

In the last decade, many electrochemical methods were developed as complementary assays thus allowing the antioxidants evaluation based on their net electric charge measurement [25–27]. Unfortunately, it is mandatory to dilute real samples that are a complex matrix of compounds that can possibly undergo oxidation to minimize the signal-to-noise ratio due to unwanted contribution to the background current [28, 29]. Most species are readily oxidized at relatively low potential on platinum, whereas, the oxidation potential for the glassy carbon electrode is higher, thus enabling the undesirable co-oxidation. Also, precaution should be taken when selecting the dilution ratio to avoid the nonlinear response. Another impediment is that during the electrochemical process, the reduced and oxidized species may be adsorbed strongly on the surface of many classical electrode types, such as graphite or carbon paste electrodes [29–32].

Screen-printed electrodes (SPEs) are novel devices developed in the last five years, fact explaining why the electrochemical approaches using microsensors are less explored. Electroanalytical methods, using SPE, present many advantages such as speed, low cost, simplicity, and low consumption of reagents when compared to other methods.

The objective of the present work was to develop the electrochemical CUPRAC method by using a screen-printed electrochemical sensor with carbon as the working and counterelectrode, as a novel device for the investigation of the antioxidant capacity. An important advantage of the SPE with carbon as the working and counterelectrode is that the thin layers of carbon-dispersed particles acting as main components of working electrode (WE) and auxiliary electrode (AE) can oxidize electroactive species at low potential that minimizes the background current and hence favour the signal-to-noise ratio. Also, the working technique was selected to minimize the adsorption process of reduced/oxidized species on the surface of electrodes.

## 2. Materials and Methods

**Figure 1:**
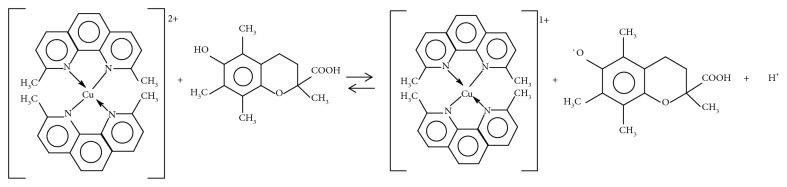
Scheme of the redox reaction between copper-neocuproine complex and trolox.

There are many indirect methods measuring the antioxidant activity of tea extracts [4, 5], monitoring the inhibition of oxidation of a certain substrate using the extracts with antioxidant properties [6]. Fundamentally, the principle of the method involves the reduction of the copper-neocuproine complex [Cu(Nc)_2_]^2+^ by the antioxidant (AOX) in the presence of ammonium acetate to form copper-neocuproine complex [Cu(Nc)_2_]^+^, a yellow compound (Figure 1), with a maximum absorption at *λ* = 450 nm [18].

### 2.1. Chemicals and Reagents

For the preparation of both standard solutions and samples, the following chemicals and reagents were used: ammonium acetate (solid); neocuproine (solid); copper chloride, CuCl_2_ × 2H_2_O (solid); and ethanol (p.a.), as well as trolox, 6-hydroxy-2,5,7,8-tetramethylchroman-2-carboxylic acid (solid).

All supporting solutions were prepared using analytical grade reagents and purified water from a Millipore Milli-Q system (conductivity ≤ 0.1 *μ*S·cm^−1^) (Millipore S. A., Molsheim, France), in accordance with well-established procedures.

### 2.2. Preparation of CUPRAC Standard Solutions

A freshly prepared buffer solution of ammonium acetate (1.2 mol·L^−1^) was tested with a Hanna HI 9125 pH meter with HI 1230B electrode and adjusted to pH = 7 by adding small amounts of 1 mol·L^−1^ NaOH. The aqueous solution of copper chloride (0.012 mol·L^−1^) was prepared with deionized water, while neocuproine (0.001 mol·L^−1^) and trolox (0.001 mol·L^−1^) were alcoholic solutions.

The standards for the calibration curve were prepared with 0.2, 0.4, 0.6, 0.8, and 1.0 mL, respectively, of the 0.001 mol·L^−1^ trolox solution, diluted with deionized water up to 4 mL, then completed to 10 mL total mixture with 2 mL from each of the following solutions: ammonium acetate buffer (1.2 mol·L^−1^), copper chloride (0.012 mol·L^−1^), and neocuproine (0.001 mol·L^−1^). The standard mixtures were maintained for 30 minutes to react; then, the absorbance of each trolox standard versus blank was measured at 450 nm wavelength.

### 2.3. Instrumentation

The absorbance measurements were recorded with a T90 + UV/VIS Spectrometer (PG Instruments Ltd.).

Electrochemical experiments were performed with a potentiostat/galvanostat/EIS analyzer Palmsense 4® integrated with the PS Trace 5® software, Version 5.3.1127 Build 198586t. A Palm Speholder assured connection with the BVT-AC1.W4 R1 (BVT Technologies) screen-printed electrodes (of 7 by 25 mm dimensions and ceramic alumina as support material) with carbon as the working and counterelectrode (Ø of the disc WE = 1 mm, WE geometric area = 0.79 mm^2^) and Ag/AgCl as the reference electrode.

### 2.4. Electrochemical and Spectrophotometric Measurements

The spectrophotometric measurements were recorded at room temperature (21 ± 1°C), by using quartz cells of a 1 cm optic path to measure the absorbance of the sample versus blank at 450 nm wavelength. For the spectrum recording, each trolox standard and sample were tested separately using a one-cell measurement at the abovementioned same spectrophotometer.

The experimental conditions for cyclic voltammetry versus open-circuit potential (CV vs OCP) were as follows: *t*_equilibration_ = 1 s, *E*_begin_ = 0 V, *E*_vertex1_ = 0 V, *E*_vertex2_ = 1 V, *E*_step_ = 0.05 V, scan rate = 0.05 V/s, number of scans = 1, *E*_begin_ vs OCP (*t*_max._ OCP = 1 s, stability criterion = 1.0 m·Vs^−1^).

For DPV, the pretreatment settings *E* condition = −0.6 V, *t* condition = 0 s, *E* deposition = −0.5 V, and *t* deposition = 0 s. DPV settings: *t*_equilibration_ = 1 s, *E*_begin_ = 0 V, *E*_end_ = 0 V, *E*_step_ = 0.005 V (*E*_pulse_ = 0.0025 V, *t*_pulse_ = 0.05 s), and scan rate = 0.025 Vs^−1^.

For both CV vs OCP and DPV, the corresponding current of the oxidation peak was used to quantify the concentration of [Cu(Nc)_2_]^1+^.

CV vs OCP curves have been modified by multiplying (30 times) and very high smoothing (25 times).

DPV curves were processed through baseline subtraction, respectively, and linear baseline followed by moving average baseline (windows size = 2 points; max. number of sweeps = 1001).

### 2.5. Practical Application

The practical application was focused on the analysis of the antioxidant capacity of various tea infusion samples (black and green) that involved simple preliminary preparation.

### 2.6. Sample Preparation

Five of each black (B1–B5) and green (G1–G5) tea samples of different brands were purchased from the town's commercial network. The infusions were made by adding 0.5 g of packed tea into 10 ml of deionized water at 95°C and maintained there for 5 minutes. After removing the satchel, the resulting infusion was let to cool down at room temperature before the preparation of the CUPRAC samples mixture, as follows: 4 ml infusion plus 2 ml of each buffer, CuCl_2_ and neocuproine solutions, to a total of 10 ml final volume.

## 3. Results and Discussion

### 3.1. Voltammetric Results

**Figure 2:**
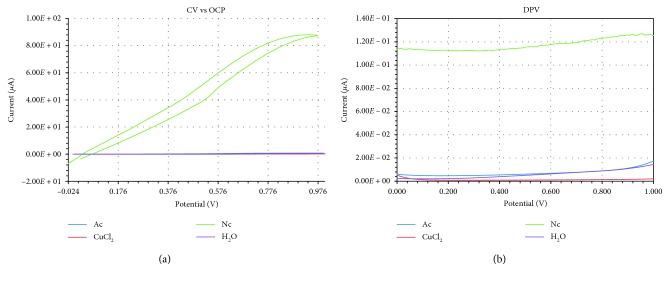
Voltammetric behaviour of each reagent of the reaction system: (a) cyclic voltammetry versus open circuit potential tests; (b) differential pulse voltammetry tests.

The electrochemical measurements were performed individually for each reagent by cyclic voltammetry versus open-circuit potential tests (Figure 2(a)) as well as by differential pulse voltammetry tests (Figure 2(b)) in order to evaluate the voltammetric behaviour of each reagent of the reaction system. Both CV vs OCP and DPV voltammograms from Figure 2 show that neocuproine has a significant bigger contribution to the electrical current signal when compared to the other reagents of the reaction's system, respectively, CuCl_2_, acetate, and water.

**Figure 3:**
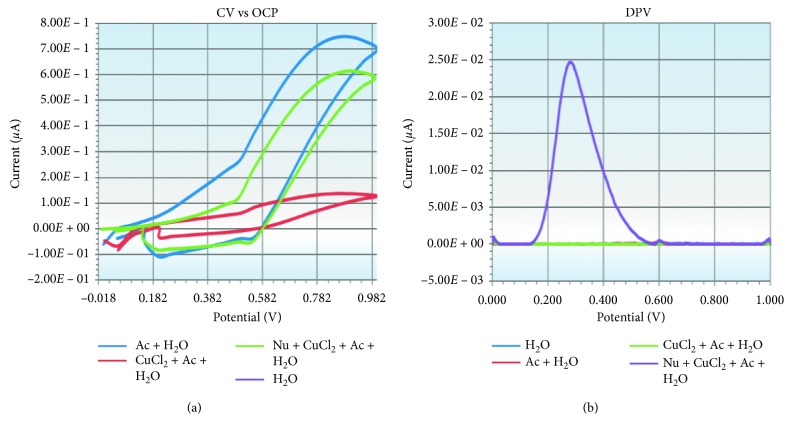
Voltammetric behaviour of the reaction system's mixture of reagents, evolution according to the working protocol: (a) cyclic voltammetry versus open circuit potential tests; (b) differential pulse voltammetry tests.

Figure 3 presents the voltammetric behaviour of the reaction system's evolution, to evaluate the electrochemical characteristics of the “blank reagents mixture” of the CUPRAC system. Practically, CV vs OCP (Figure 3(a)) and DPV (Figure 3(b)) voltammograms were recorded upon each reagent addition into the reaction system, according to the working protocol starting with the mixture of water and acetate, mixture of water, acetate, and CuCl_2_, and finally the mixture of water, acetate, CuCl_2_, and neocuproine.

The cyclic voltammograms (Figure 3(a)) show that the mixture of CuCl_2,_ acetate, and water induced a reversible redox process (Cu^2+^↔Cu^1+^) with a low current corresponding to a quasi-equal reduction and oxidation peak. The mixture of neocuproine, CuCl_2,_ acetate, and water caused the change of the redox potential of the system (Cu^2+^↔Cu^1+^), by increasing with 0.3 V the oxidative capacity of the reaction's couple (Cu^2+^↔Cu^1+^). A change in the redox potential of the CUPRAC system of about 0.4 V was explained by Cárdenas et al. [21, 22], considering Nernst equation and the kinetics determinants of the redox (Cu^2+^↔Cu^1+^) system. Thus, comparative to the significant oxidation peak current showed by the DPV voltammogram of neocuproine, CuCl_2,_ acetate, and water mixture (Figure 3(b)), the mixture of CuCl_2,_ acetate, and water, as well as the mixture of ammonium acetate and water (the support electrolyte solution), does not present significant redox peaks for the potential variations between 0 and 1 V.

**Figure 4:**
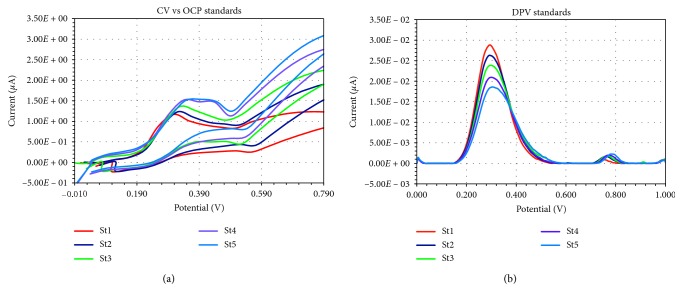
Voltammograms of trolox standards: (a) cyclic voltammetry versus open circuit potential tests; (b) differential pulse voltammetry tests.

The voltammograms obtained through cyclic voltammetry versus open-circuit potential for the trolox standards were processed by 30 times multiplying and smoothing with PS Trace 5 software. CV vs OCP voltammograms of cyclic voltammetry versus open-circuit potential tests (Figure 4(a)) showed that trolox addition determined the shifting of the open-circuit potential towards more negative values. Similarly, with the data reported by Cárdenas et al. [21, 22], these results may be explained with Nernst equation as a consequence of the increasing ratio between the main system's reducing [Cu(Nc)_2_]^1+^ and oxidant [Cu(Nc)_2_]^2+^ species. Trolox addition induced an oxidation peak (without a previous reduction process), as once the antioxidant is added, the generated [Cu(Nc)_2_]^1+^ species will be electrochemically oxidized and the oxidation current will be registered. Consequently, the oxidation peak current is expected to be proportional with the amount of added trolox. Thus, the measured current values corresponding to the oxidation peaks generate the CV vs OCP standard calibration curve.

**Figure 5:**
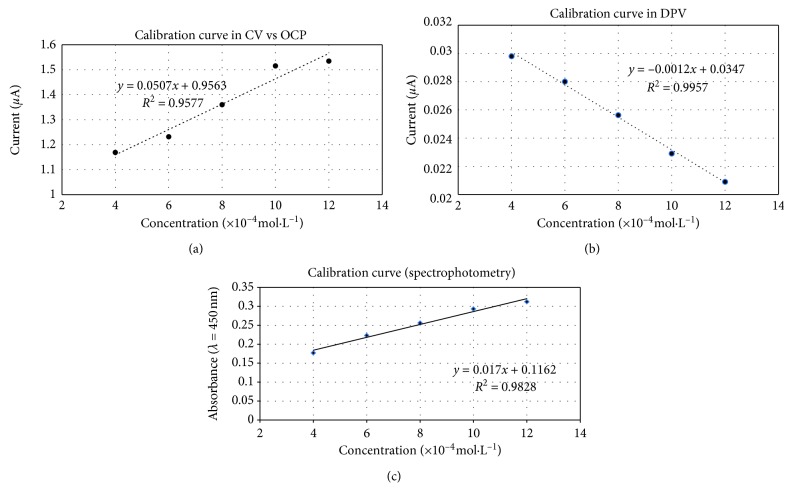
Calibration curve for trolox standards: (a) cyclic voltammetry versus open circuit potential tests; (b) differential pulse voltammetry tests; (c) spectrophotometric tests.

The voltammograms obtained through cyclic voltammetry versus open-circuit potential and differential pulse voltammetry for the trolox standards (Figures 4(a) and 4(b)) were processed by multiplying and smoothing (CV vs OCP) or linear/moving baseline extraction (DPV), and the maximum registered values for the current expressed in *μ*A were considered to generate the standard calibration curve (Figures 5(a) and 5(b)). A basic comparison between CV vs OCP and DPV voltammograms from Figure 4 highlights that the DPV technique allowed a better response, since as the antioxidant capacity determined with DPV increases, the data obtained from CV vs OCP results present the tendency to reach a plateau. These results may be explained considering that DPV analysis is able to differentiate against the blocking of the electrode surface by the adsorbing antioxidant species present in the electrolyte solution that may determine the subsequent decreasing number of the active sites on the surface of the screen-printed microelectrodes [33].

Both cyclic voltammetry versus open-circuit potential results as well as differential pulse voltammetry results were fitted to a linear function (Figures 5(a) and 5(b)). Differential pulse voltammetry data presented an excellent linear fit (*R*^2^ > 0.996) while cyclic voltammetry versus open-circuit potential showed a very good linear fit (*R*^2^ > 0.957). The absorbance's measurements of trolox standards were conducted exactly 30 minutes after preparation. Similar to electrochemical data, the spectrophotometric results fitted a linear function (*R*^2^ > 0.9828) between 4 and 12 · 10^−4^ mol·L^−1^. These results suggest that it is more feasible to employ differential pulse voltammetric measurements based on CUPRAC reaction to determine antioxidant capacity as an alternative to the classical spectrophotometric measurements.

Both DPV and spectrophotometric methods were investigated for precision, limit of detection, and limit of quantification.

Precision, as a measure of statistical variability, was investigated for the standard solution corresponding to the 12 · 10^−4^ mol·L^−1^ trolox concentration under two aspects: repeatability and intermediate precision. Repeatability results (expressing the consistency of the measurements under identical experimental conditions at short time intervals, in the same day/intra-assay), as well as intermediate precision (expressing the fidelity of the measurement at large intervals of time, in different days/interassays), are presented in Table 1. Both types of precision tests framed into the recommended acceptance criteria for relative standard deviation (RSD) ≤ 2%, for either spectrophotometric or DPV measurements.

**Figure 6:**
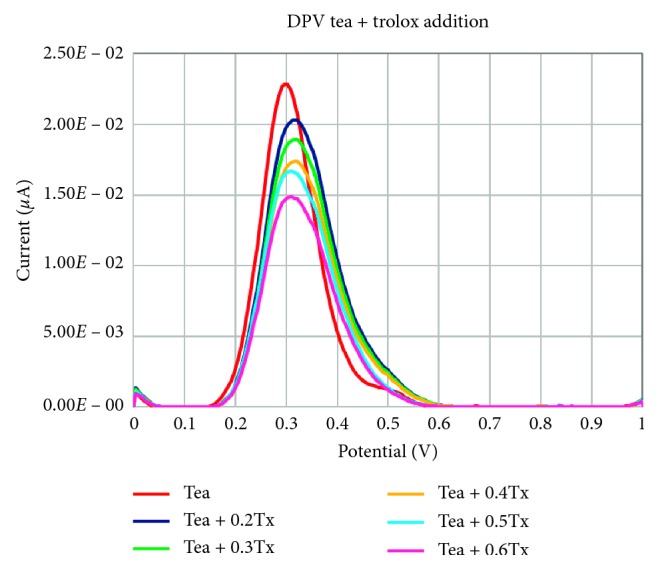
Voltammograms of trolox standard additions to a tea sample.

To confirm the precision of the proposed methods, trolox standard addition was applied to a tea sample. The resulted voltammograms of trolox standard additions to a tea sample measured by DPV are presented in Figure 6.

**Figure 7:**
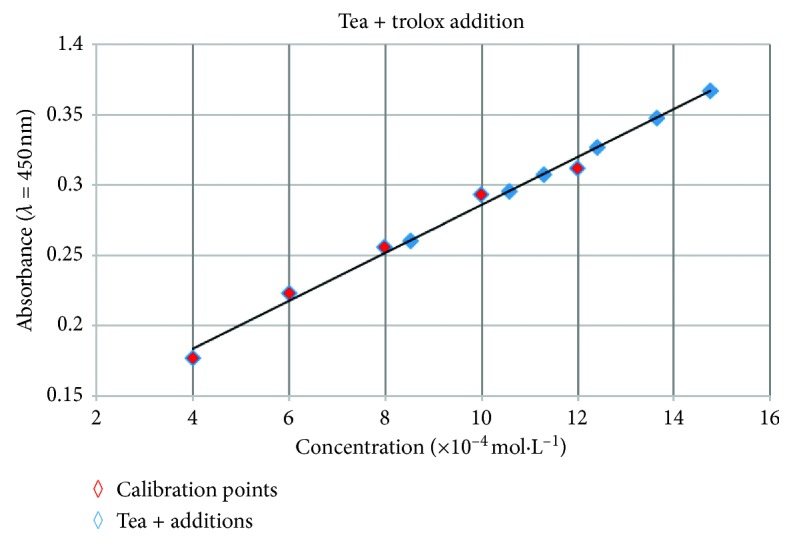
Spectrophotometric calibration curve of trolox standards + trolox standard addition to a tea sample.

Spectrophotometric calibration curve of trolox standards (red points) and the corresponding trolox standard addition to a tea sample (blue points) are shown in Figure 7.

As Table 2 presents, the recovery rates for different trolox additions within the investigated range varied between 97.7 and 101.9% for DPV and between 95.55 and 103.9% for spectrophotometry.

To evaluate the limit of detection (LOD), the lowest trolox standard concentration was diluted serially and analysed six times with each technique as well. Then, the mean and the standard deviation and relative standard deviations were calculated (Table 3). Limit of quantification (LOQ) is practically the lowest concentration in the standard curve (4 · 10^−4^ mol·L^−1^) that could be quantified (determined) with acceptable precision and accuracy under the same experimental conditions (the lowest concentration in the standard curve). Both limit detection tests framed into the recommended acceptance criteria for relative standard deviation (RSD) ≤ 10%, for either spectrophotometric or DPV measurements.

**Figure 8:**
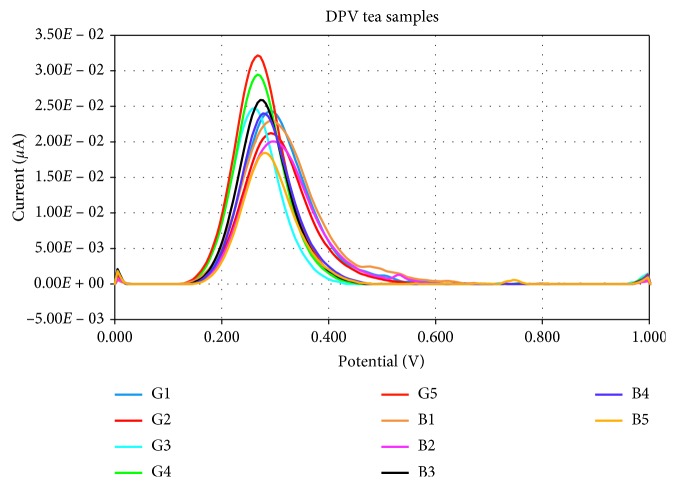
Voltammograms of tea samples by differential pulse voltammetry tests.

The tea sample voltammograms (Figure 8) showed that DPV technique registered a proper response, which demonstrates that the working parameters of this voltammetric method as well as the preprocessing tea samples protocol were optimally chosen.

The voltammograms of the tea samples were used for the calculus of the antioxidant capacity expressed in trolox equivalents, as presented in Table 4.

The results from Table 4 sustain that black tea samples presented higher antioxidant capacity than green ones, which agrees with other reported results focusing similar researches [30, 31].

**Figure 9:**
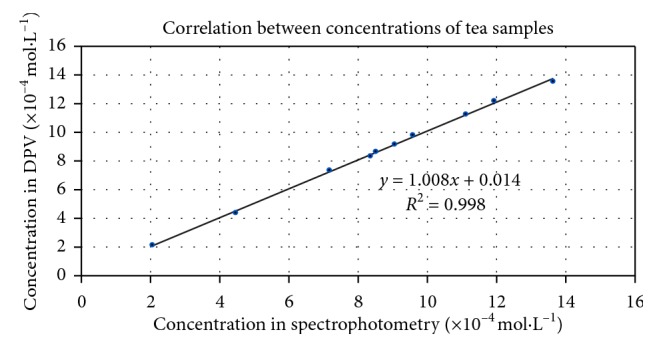
Correlations of tea sample concentrations between differential pulse voltammetry and spectrophotometry tests.

To evaluate the efficiency of the voltammetric method used in quantifying the concentrations of the samples, correlation between differential pulse voltammetry and spectrophotometry data was made (Figure 9). Analytical results of the antioxidant capacity (expressed as mol·L^−1^ trolox equivalents) of the tea samples showed a good agreement in the case of spectrophotometry and differential pulse voltammetry (*R*^2^ > 0.998).

**Figure 10:**
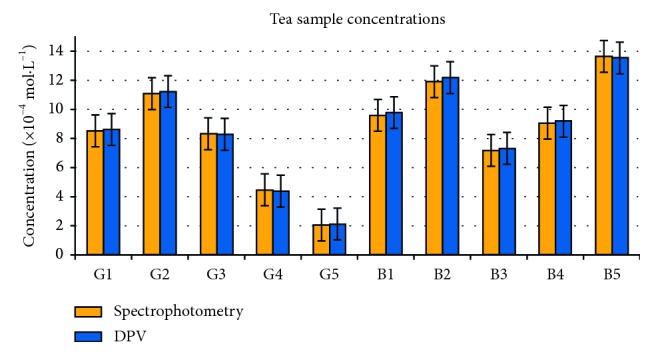
Comparative results for the tea infusion samples.

However, the antioxidant capacity (trolox equivalents) of the tea infusion samples ranged between 2.04 and 11.23 · 10^−4^ mol·L^−1^ for green tea samples and 7.16–13.63 · 10^−4^ mol·L^−1^ for black tea samples (Figure 10), with values that are fitting into the normal domain of the variability of similar researches [4, 6, 8, 11, 18, 20–22, 30–32, 34] that signal variations mainly depending on the specific peculiarities of the tea variety and also on the extraction method that was used.

However, the statistic tests (Table 5) showed no significant (*P* > 0.05) differences between the average values (*t*-test) as well as between standard deviation values (*F*-test) of the spectrophotometric and DPV results.

## 4. Conclusions

The analysis from the present study were employed to find an alternative to classical CUPRAC spectrophotometric or electrometric methods and to quantify the antioxidant capacity by using voltammetric technique with screen-printed microelectrodes. The screen-printed microelectrodes response was linearly correlated with Trolox content of the standards. Analytical results of the antioxidant capacity (expressed as mol·L^−1^ Trolox equivalents) of the tea samples showed a better agreement in the case of spectrophotometry and differential pulse voltammetry (*R*^2^ > 0.998).

## Figures and Tables

**Table 1:** Precision measurements for 12 · 10^−4^ mol·L^−1^ trolox standard solution.

Statistical parameter	Intra-assay						Interassay	
	DPV			Spectrophotometry			DPV	Spectrophotometry
	Day 1	Day 2	Day 3	Day 1	Day 2	Day 3		
Average	12.244	11.890	12.110	12.439	11.969	12.204	12.081	12.204
Standard deviation (SD)	0.130	0.128	0.042	0.189	0.0334	0.170	0.179	0.235
Relative standard deviation (RSD)	1.061	1.073	0.348	1.520	0.284	1.391	1.482	1.928

**Table 2:** Recovery rates for trolox standard addition to a tea sample.

Sample	Trolox added (×10^−4^ mol·L^−1^)		Trolox found (×10^−4^ mol·L^−1^)		Recovery (%)	
	DPV	Spectrophotometry	DPV	Spectrophotometry	DPV	Spectrophotometry
Addition 1	2	2	1.954	2.059	97.692	102.941
Addition 2	3	3	3.023	2.864	100.769	95.466
Addition 3	4	4	4.077	3.882	101.925	97.059
Addition 4	5	5	4.958	5.118	99.160	102.353
Addition 5	6	6	6.130	6.235	101.166	103.922

**Table 3:** Limit of detection (LOD) and limit of quantification (LOQ) in DPV and spectrophotometry.

Method	DPV	Spectrophotometry	DPV	Spectrophotometry
Statistical parameter	Limit of detection (LOD)		Limit of quantification (LOQ)	
Trolox concentration	2 · 10^−4^ mol·L^−1^		4 · 10^−4^ mol·L^−1^	
Average (mean of 6 repetitions)	1.996	4.047	4.015	2.037
SD	0.175	0.363	0.276	0.176
RSD	8.742	8.960	6.863	8.646

**Table 4:** Current values (*μ*A), absorbance values, and the corresponding concentration values (×10^−4^ mol·L^−1^) of the tea samples.

Method	DPV		Spectrophotometry	
Sample	Current (*μ*A)	Concentration (×10^−4 ^mol·L^−1^)	Absorbance (*λ* = 450 nm)	Concentration (×10^−4 ^mol·L^−1^)
G1	0.024351	8.624014	0.261	8.517647
G2	0.021224	11.230403	0.305	11.10588
G3	0.247403	8.299720	0.258	8.341176
G4	0.029478	4.351879	0.192	4.458824
G5	0.032166	2.111386	0.151	2.047059

B1	0.022960	9.783305	0.279	9.576471
B2	0.020059	12.200286	0.319	11.92941
B3	0.025903	7.330995	0.238	7.164706
B4	0.023679	9.183913	0.270	9.047059
B5	0.018433	13.555708	0.348	13.63529

**Table 5:** *t*-test and *F*-test results of tea sample concentration values.

Test	*P* value	Statistical results
*t*-test	*P*=0.954 *P*(*x* ≤ *t*)=0.477	*t* = 0.0579642 is in the 95% critical value accepted range: (−2.1009 to 2.1009) *X*_G1_ − *X*_G2_=0.085 is in the 95% accepted range: (−3.0700 to 3.0700)

F-test	*P*=0.979 *P*(*x* ≤ *f*)=0.489	*f* = 0.982471 is in the 95% critical value accepted range: (0.2484–4.0260) SD_G1_/SD_G2_ = 0.99 is in the 95% accepted range: (0.4980–2.0060)

## Data Availability

The raw data used to support the findings of this study are available from the corresponding author upon request.
